# Lunacy Reform

**Published:** 1922-07

**Authors:** H. J. Norman

**Affiliations:** (Lecturer on Mental Diseases, Westminster Hospital, Senior Assistant Medical Officer at Camberwell House)


### LUNACY REFORM.

**By H. J. NORMAN, M.B., Ch.B. (Lecturer on Mental Diseases, Westminster Hospital, Senior Assistant Medical Officer at Camberwell House).**

UNDER this title there has recently been issued the report of the proceedings at a conference convened by Sir Frederick Willis, Chairman of the Board of Control, at which there were present Commissioners of the Board, Chairmen of Visiting Committees and Medical Superintendents of County and Borough Mental Hospitals. They met to consider " in what directions lunacy administration and the treatment of persons suffering from mental disease may be improved." The discussions which took place show the lively interest taken in such matters by those primarily responsible for the care and control of the insane. There was, of course, a general agreement as to the need still further to improve matters; difference of opinion, where it was apparent, was rather as to detail and as to the most efficient methods to be adopted.

As Sir Alfred Mond wisely pointed out in his introductory speech, the financial aspect of the problem must not be lost sight of; at the same time, " we have always to bear in mind what I may call the relation of proportion. Economy does not always consist in spending no money." It is obviously useless to advocate at the present period of financial stringency reforms which are likely to entail large capital expenditure. We may at once rule out of consideration at the moment suggestions as to the scrapping of existing buildings and other rather wild schemes which have not lacked advocates.

The early treatment of mental disorders was one of the subjects first discussed. The formation of psychiatric clinics is one method whereby the patient may be brought into contact with the skilled physician. But the out-patient treatment of such cases presents greater difficulties than do ordinary medical and surgical disorders. Many cases, even in the incipient stage, need to be treated away from home, and often the removal of the patient from domestic worries is the first step towards cure. The absence of irritating stimuli is as essential to the disordered brain as it is to, let us say, the inflamed eye or the dyspeptic stomach. Without the possibility of in-patient treatment, such clinics can be of only limited utility. A general hospital is not the best place for such treatment, except for certain cases of transient excitement or delirium—such as delirium tremens, for example, or acute confusional cases. And here it may be explicitly stated that the suggestion that there should be no association of any kind with " lunacy " is dangerous in theory and impossible—if the best result are to be obtained—in practice. There is a too prevalent idea at the present time that any cheerful and enthusiastic amateur can do more for the mental patient than can those who have devoted years to the study and care of the insane. We must not blind ourselves to the fact that what we call insanity is a disorder which shows itself in all degrees, from the mild, " borderland " condition to one in which the symptoms are of the most pronounced character. It is only necessary to look at the newspaper on practically any day of the week to see how some of the—apparently—slight melancholic disorders end—in a verdict of " Suicide while of unsound mind." Many valuable lives would be saved if relatives—and medical men—could be brought to realise the risk they incur by procrastinating in cases where suicidal impulses are present. There are even doctors who undertake the treatment of mental patients, and who—through lack of adequate training—fail to discriminate between the hypochondriac or the neurasthenic and the true melancholic—with disastrous results.

The early treatment of patients with means is already very much an accomplished fact. There are hundreds of nursing-homes and private houses throughout the country where they are received. Even in this respect, however, matters are not satisfactory. Many of those who receive patients in this way are—if the law is strictly observed—acting illegally; for if a patient is technically of unsound mind, even for the briefest period, the person who receives him for payment is committing an offence, and is liable to prosecution. This is an intolerable state of affairs. The opinion of the Conference was that at the earliest possible moment a short Bill should be introduced into Parliament to alter the law on this point, and to allow a probationary period before it is necessary to resort to certification. As a matter of fact, this would probably have been done some two years ago had not this suggested amendment of the Lunacy Act been made to suffer for the sins of the very miscellaneous provisions of the Bill then under discussion. But if any relaxation of the law is allowed, there must be adequate inspection of such homes, whether or not a charge is made by those who control them; and some standard of efficiency should be set for those who undertake the nursing of any mental patients. The Conference agreed that the central department which should exercise this supervision ought to be the Board of Control. There is no weight of opinion opposed to this most reasonable suggestion. The appointment of another committee of officials would not satisfy the hypercritical—at any rate, for more than a short time; and it would inevitably lead to a greatly increased expenditure. (It is, of course, very probable that the Board of Control will have to appoint a certain number of inspectors, but this is quite another matter from instituting another Government sub-department.) The duties of separate committees too, would very likely overlap, and there would be either friction between the two or duplication of work. It would be better, if changes are to be made, to bring about a closer amalgamation of the two existing bodies, the Board of Control and the Lord Chancellor's Visitors. It is obvious that these officials are, let it be added, moving with the times, and are not advocating reactionary methods. The Report of the doings of this Conference is in itself strong evidence that this is so.

To return to the clinics, there should be some arrangement whereby poorer patients might avail themselves of skilled advice, either at the general hospitals, at the infirmaries, or at a special department in connection with or in the vicinity of the mental hospitals, such as that which is being conducted by the authorities of Bethlem Hospital. Beds for the immediate treatment of some of the cases should be provided. In addition, there should be nursing homes or hostels to which suitable cases could be sent as soon as possible, and where patients could be treated gratuitously or for a small payment. Dr. Bedford Pierce mentioned such homes as having been tried in connection with the " Retreat " at York, but was not able to speak very highly of the results so far obtained. Still, such homes need not involve large capital expenditure ; and the fact that they do already exist for well-to-do patients seems to prove that the scheme is a workable one.

Even with such establishments provided, the difficulties as to early treatment would not be overcome. Quite a large proportion of the cases show within a very short time of the onset of the disorder such acute symptoms that not only are they unable to decide that they require treatment, but they are too restless and noisy for admission to a small home. They require institutional care, and there is no reason why they should not have it without any necessity for certification. Often they are young people who are temporarily unhinged, and who may be so much better in a few weeks that no one would think of certifying them as insane. If there is a proper system of notification and supervision, the step may safely be taken of depriving them of their liberty even without their consent. As a matter of fact, this is already done when such patients are taken *nolens volens* to the infirmary.

Another matter which is cognate to this is the necessity that arrangements should be made for the admission of voluntary boarders to the public asylums. This is already possible in so far as the registered hospitals and the private asylums or licensed houses are concerned, and quite a large number of such patients are admitted to these establishments every year. In spite of popular prejudice against voluntary patients associating with those who are certified, there is no evidence that such association is bad for them. Indeed, experience goes to prove quite the opposite. However this aspect of it may be regarded, there is no doubt as to the advisability of giving patients the opportunity of placing themselves under care in any mental hospital if they choose to do so.

